# Sentinel node mapping for post-endoscopic resection gastric cancer: multicenter retrospective cohort study in Japan

**DOI:** 10.1007/s10120-019-01038-3

**Published:** 2020-01-11

**Authors:** Shuhei Mayanagi, Naoto Takahashi, Norio Mitsumori, Takaaki Arigami, Shoji Natsugoe, Yoshihisa Yaguchi, Takeshi Suda, Shinichi Kinami, Masaki Ohi, Hirofumi Kawakubo, Yasunori Sato, Hiroya Takeuchi, Takashi Aikou, Yuko Kitagawa, Japanese Society for Sentinel Node Navigation Surgery

**Affiliations:** 1 Department of Surgery, Keio University School of Medicine, 35 Shinanomachi, Shinjuku-ku, Tokyo, 160-8582 Japan; 2 Department of Surgery, The Jikei University, School of Medicine, Tokyo, Japan; 3 Department of Digestive Surgery, Breast and Thyroid Surgery, Kagoshima University Graduate School of Medical and Dental Sciences, Kagoshima, Japan; 4 Department of Surgery, National Defense Medical College, Tokorozawa, Japan; 5 Department of Gastrointestinal and Pediatric Surgery, Tokyo Medical University, Tokyo, Japan; 6 Department of Surgical Oncology, Kanazawa Medical University, Ishikawa, Japan; 7 Department of Gastrointestinal and Pediatric Surgery, Division of Reparative Medicine Institute of Life Sciences, Mie University Graduate School of Medicine, Tsu, Mie Japan; 8 Department of Preventive Medicine and Public Health, Biostatistics at Clinical and Translational Research Center, Keio University School of Medicine, Tokyo, Japan; 9 Department of Surgery, Hamamatsu University School of Medicine, Hamamatsu, Japan; 10 Seiunkai Hospital, Kagoshima, Japan

**Keywords:** Early gastric cancer, Endoscopic mucosal resection, Endoscopic submucosal dissection, Sentinel lymph node

## Abstract

**Background:**

Standard gastrectomy with systematic lymphadenectomy as an additional surgery after endoscopic resection (ER) causes a deterioration in long-term quality of life. If the sentinel lymph node (SN) basin concept can be applied in post-ER gastric cancer, minimal surgery can be applied without reducing the curability. This retrospective multicenter cohort study aimed to verify the validity of the SN basin concept in post-ER gastric cancer.

**Patients and methods:**

Individual data of 132 patients who underwent SN mapping after ER were collected from 8 university hospitals in Japan from 2001 to 2016. Tracers were injected endoscopically in the submucosal layer at four sites around the post-ER scar. We compared the SN basin distribution of post-ER gastric cancer with that of 275 patients with non-ER gastric cancer.

**Results:**

Two cases of SN were unidentified, both involving a single tracer (SN detection rate: 98.5%). Nine cases (6.8%) of lymph node metastasis were found, of which eight had a metastatic lymph node within the SNs and one had a non-SN metastasis within the SN basin. The diagnostic sensitivity of SN mapping for lymph node metastasis was 88.9% in post-ER group and 95.7% in non-ER group (*P* = 0.490); the accuracy was 99.2% and 99.6% (*P* = 0.539), respectively. Regarding the SN basin, no significant intergroup differences were found regardless of the primary tumor location.

**Conclusions:**

Our findings clarified the feasibility of SN mapping based on the SN basin concept in patients with gastric cancer who previously underwent ER.

## Introduction

The population of patients with gastric cancer is currently aging in Japan. In 2017, 45.6% of patients with newly diagnosed gastric cancer were > 75 years old [1]. In such a patient background, the popularity of endoscopic resection (ER), including endoscopic mucosal resection (EMR) [2] and endoscopic submucosal dissection (ESD) [3, 4], as a less invasive treatment modality for early gastric cancer has spread dramatically. It is particularly useful for organ preservation and maintaining quality of life (QOL) after treatment. According to the Japanese guidelines for gastric cancer treatment [5], lesions with < 1% estimated risk of lymph node metastasis are considered equivalent to surgical gastrectomy and defined as absolute-indication lesions. In addition, although the risk of lymph node metastasis is estimated to be < 1%, lesions for which long-term prognostic evidence is poor are defined as expanded-indication lesions. Lesions that were initially defined as expanded-indication have become absolute-indication lesions as verified in a large-scale clinical trial such as JCOG0607 [6]. The indications for ER are gradually expanding. ER curability is determined by two factors: extent of local resection and the possibility of lymph node metastasis. Additional surgical resection, usually laparoscopic standard gastrectomy with systematic lymphadenectomy, is the standard treatment if the risk of lymph node metastasis is estimated from the resected specimen after ER. However, in a few cases, additional surgical resection did not show tumor remnants or lymph node metastasis. Moreover, because of standard gastrectomy and systematic lymphadenectomy, it becomes a serious problem that causes a decrease in long-term QOL, including postoperative weight loss, especially in elderly patients.

The SN concept supports the theory that sentinel lymph nodes (SNs) receive lymph flow directly from the tumor and experience the first lymph node metastasis. If no lymph node metastasis is found in the SNs, it is judged that there is no metastasis in other lymph nodes. Surgery using the SN concept is actively attempted as a safer attempt at minimally invasive surgery. In breast cancer and malignant melanoma, individualized surgery, including SN biopsy, has been standardized [7, 8], and the application to clinical practice for various cancers has also been reported [9–12]. In early gastric cancer, Miwa et al. proposed the concept of SN basin dissection in which SN basins contained true metastatic nodes even in patients with a false-negative SN biopsy [13]. A prospective multicenter clinical trial was conducted by our study group, the Japanese Society for Sentinel Node Navigation Surgery (SNNS), in order to verify the validity of SN mapping for gastric cancer [14]. This prospective study determined that SN basin dissection, which was considered a minimally focused lymphadenectomy, was acceptable without compromising the curability of cT1 N0 gastric cancer with no previous treatment. If the SN basin concept can be applied even in post-ER lesions, it may be possible to conduct minimal surgery that preserves postoperative QOL without degrading the curability. A detection of lymph node metastasis by SN biopsy followed by SN basin resection as an additional surgery for post-ER gastric cancer is a highly attractive treatment strategy. However, the lymph flow around the primary lesion may change by ER, and this has not been verified in previous prospective study. Regarding the SN of post-ER gastric cancer, only two single-center retrospective studies had high SN detection rates and diagnostic accuracies for lymph node metastasis [15, 16]. The SN basin reportedly did not differ from the control group without ER. However, single-center studies include small sample sizes, and inadequate evidence supports its validity. Therefore, this multicenter retrospective cohort study aimed to verify the feasibility of the SN basin concept in post-ER gastric cancer.

## Patients and methods

### Patients

From February 2001 to March 2016, the individual data of patients who underwent SN mapping after ER were collected for this retrospective study from the following eight university hospitals: Keio University, Jikei University, Kagoshima University, National Defense Medical College, Tokyo Medical University, Mie University, and Kanazawa Medical University. The conditions of the facilities for participating in this study were as follows: (1) belonging to the Japanese society of SNNS, (2) having capabilities of providing the dual-tracer method, (3) high volume center for SNNS, (4) experienced surgical staffs, and (5) special functioning hospitals approved by the Ministry of Health, Labour and Welfare in Japan. The Japanese Society for SNNS multicenter prospective trial data [14] were used as a historical control with pT1, < 4 cm in diameter, and no previous treatment. The patient data were collected after approval was obtained from the institutional review board of each hospital. (Approval numbers: Keio University 2017-0353, Jikei University 30-314(9335), Kagoshima University 180081, National Defense Medical College 2958, Tokyo Medical University T2018-0020, Mie University H2019-015 and Kanazawa Medical University 1315).

### SN mapping procedure

We identified SNs using either the radioisotope (RI) 99m Tc tin colloid or dye [indocyanine green (ICG), patent blue, or indigo carmine], or a combination of the tracers that were used in a previous clinical trial [14]. Briefly, 2.0 ml of RI (99 m Tc tin colloid; 0.5 ml × 4 points; 150 MBq; 0.3 mCi at the time of surgery) was injected endoscopically in the submucosal layer at four sites around the post-ER scar the day before surgery. It was difficult to inject the tracers in the scar tissue after ER. Therefore, the tracers were properly injected in the submucosal layer around the scar at a distance of approximately 2 cm from the center of the ER scar. At the start of the surgery, the gastrocolic ligament was divided to visualize every direction of lymph flow from the stomach. Then, 0.5 ml of each of the dye (0.5% ICG, patent blue or indigo carmine) was injected intraoperatively on four spots of the submucosa around the ER scar with the same procedure as the RI injection. Immediately after the dye injection, the lymphatic vessels and lymph nodes that were dyed blue or fluoresced under infrared observation were identified, and the lymph nodes stained up to 15 min after dye injection were regarded as SNs. At the same time, radioactive SNs were identified using a portable gamma probe. The lymph nodes with a radioactivity of > 10 times the background were also defined as SNs. In non-ER gastric cancer patients, a historical control, SN detection was performed in the same way using both RI 99m Tc and dye combination in all cases.

Gastric lymphatic basins were classified into five directions along the main gastric feeding arteries as follows: left gastric artery (lymph node stations 1, 3a, and 7), right gastric artery (lymph node stations 3b, 5, and 8a), left gastroepiploic artery (lymph node stations 4sa and 4sb), right gastroepiploic artery (lymph node stations 4d and 6), and posterior gastric artery (lymph node station 11p) [17, 18].

Intraoperative histological examinations using a frozen section of each SN were optional and performed on a case-to-case basis. After SN mapping, we performed a prophylactic lymph node dissection, including at least the lymphatic basin with the SNs.

### Statistical analyses

Baseline clinical and pathological variables were expressed as median and range for continuous variables or frequency and proportion for categorical variables. Statistical analyses were performed using the SPSS version 19 software (IBM Corporation, Armonk, NY, USA). The clinical and pathological variables were analyzed using the Chi-square and Fisher exact tests. Differences were considered statistically significant at *p* values of < 0.05.

## Results

The clinicopathological parameter values of the 132 patients who underwent SN mapping after ER are shown in Table 1. The median age of the patients was 68 years, and 80% of the patients in the ER group were male. In the ER group, the primary tumor was located in the upper, middle, and lower parts of the stomach in 27%, 45%, and 29% of the patients, respectively, with no statistically significant difference from the non-ER group (*p* = 0.137). After ER, the pathological *T* factor was deeper than T1b2 (SM2) in approximately 52% of all the patients. The median tumor diameter was 19 mm, which was smaller than that in the non-ER group. The interval from ER to SN mapping was 66 days. The surgical procedures and reasons for the additional surgery are listed in Table 2. Of all the patients, 52% underwent minimalized surgery, whereas 5% underwent only SN basin resection. Pathological *T* factor and resection margin/local recurrence occurred in approximately 50% of the patients, while lymphovascular infiltration affected approximately one-third of all the patients.

**Table 1:** Patients' characteristics

	ER (+) *N* = 132		ER (−) *N* = 275		*p* value
Age, years	68 (30–91)		62 (29–87)		< 0.001
Sex (male/female)	105/27 (80%/20%)		183/92 (67%/33%)		0.007
Location					
(in long axis of the stomach)					0.137
Upper	35	27%	56	20%	
Middle	59	45%	114	41%	
Lower	38	29%	105	38%	
(in short axis of the stomach)					0.187
Lesser curvature	51	39%	112	41%	
Greater curvature	34	26%	52	19%	
Anterior wall	14	11%	46	17%	
Posterior wall	26	20%	65	24%	
Unknown	7	4%			
*T* factor					< 0.001
pT1a	28	21%	146	53%	
pT1b1	31	22%	42	15%	
> pT1b2	68	52%	87	32%	
Unknown	5	5%			
Tumor size, mm	19 (5–40)		25 (1–40)		< 0.001
Duration from ER to SN mapping, days	66 (10–1205)				

Data are presented as median (range) or frequency and percentage

*ER* endoscopic resection, *SN* sentinel lymph node

**Table 2:** Surgical findings and factors for additional surgery

	ER (+) *N* = 132	
Surgical procedure		
Distal gastrectomy	60	45%
Total gastrectomy	4	3%
Proximal gastrectomy	23	17%
Pylorus-preserving gastrectomy	10	8%
Segmental gastrectomy	14	11%
Local resection	14	11%
SN basin resection only	7	5%
Lymphadenectomy		
SN basin (D0)	32	24%
D1	17	13%
D1+	65	49%
D2	18	14%
Factor for additional surgery		
*T* factor (deeper than SM2)	68	52%
Beyond the expanded criteria for ER [size, ulcerative findings (UL) and histological type]	11	8%
Horizontal margin/vertical margin of local recurrence	63	48%
Lymphovascular infiltration	47	36%
Other		
Non-en bloc resection	1	1%
Perforation	2	2%

*ER* endoscopic resection

Regarding the tracer for SN mapping, 68 patients (52%) received dual tracers, including both RI and dye, while 63 patients (48%) received a single tracer [dye only in 49 (37%) and RI only in 15 (11%)]. Two cases of SN were unidentified, both involving a single tracer (either RI or dye only). The primary tumor in both cases with an unidentified SN was a T1b-SM2 lesion that had been subjected to ESD. The patient who received dye only was obese and experienced a perforation during ESD. Therefore, the SN detection rate was 98.5% (130/132; Table 3).

**Table 3:** Results of SN mapping

	ER (+)		ER (−)		*p* value
Overall lymph node metastasis rate, %	6.8	(9/132)	8.4	(23/275)	0.588
Number of SN	5 (1–22)		5 (1–24)		0.820
Detection rate, %	98.5	(130/132)	100.0	(275/275)	0.105
Sensitivity, %	88.9	(8/9)	95.7	(22/23)	0.490
Negative predictive value, %	99.2	(121/122)	99.6	(252/253)	0.545
Diagnostic accuracy, %	99.2	(129/130)	99.6	(274/275)	0.539

Data are presented as median (range) or frequency and percentage

*ER* endoscopic resection

**Fig. 1:**
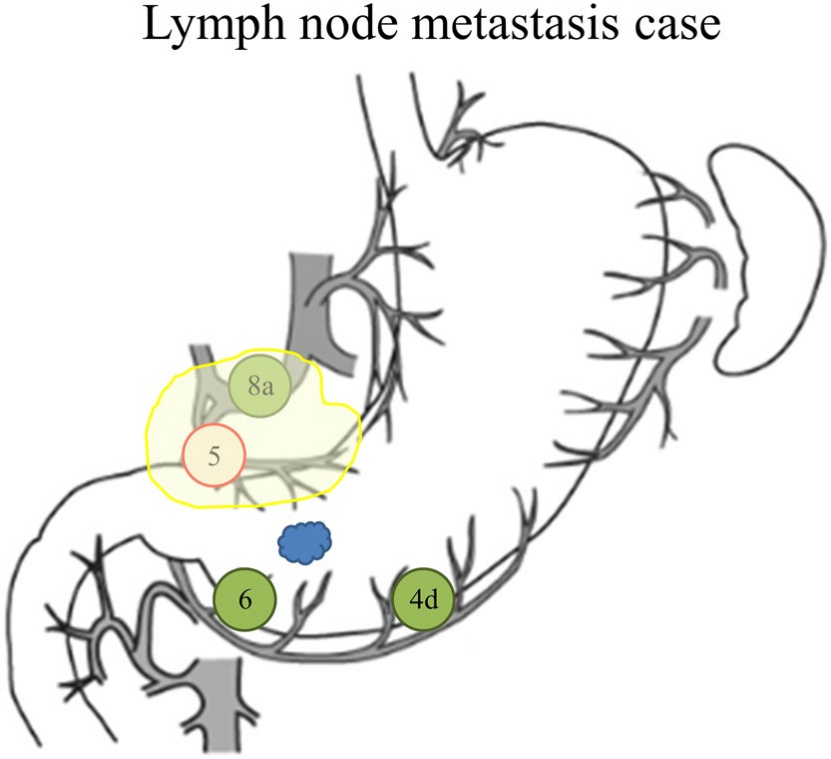
A false-negative case (L Post tub1 T1a-M ly0 v0 post-EMR, ICG only). The sentinel lymph nodes (SNs) were detected in lymph node stations 4d, 6, and 8. The metastatic lymph node was postoperatively diagnosed in lymph node station 5. Non-SN metastasis was included within the right gastric arterial basin. The yellow area indicates the right gastric arterial basin detected as one of the SN basins

Of the nine cases (6.8%) of LN metastasis, eight had metastatic LN within SNs and one had non-SN metastasis within a SN basin. In the single false-negative case, the primary tumor with pT1a that was located at the posterior wall of the lower third of the stomach was resected using EMR. SN mapping was performed using a single tracer with ICG at 6 months after the EMR. Seven SNs were detected in lymph node stations 4d, 6, and 8; however, the metastatic lymph node was postoperatively diagnosed in station 5 (Fig. 1). Therefore, a non-SN metastasis was included within the right gastric arterial basin detected as the sentinel basin. For the patients with gastric cancer who had undergone ER, the diagnostic sensitivity and accuracy of SN mapping for lymph node metastasis were 88.9% and 99.2%, respectively (Table 3).

**Fig. 2:**
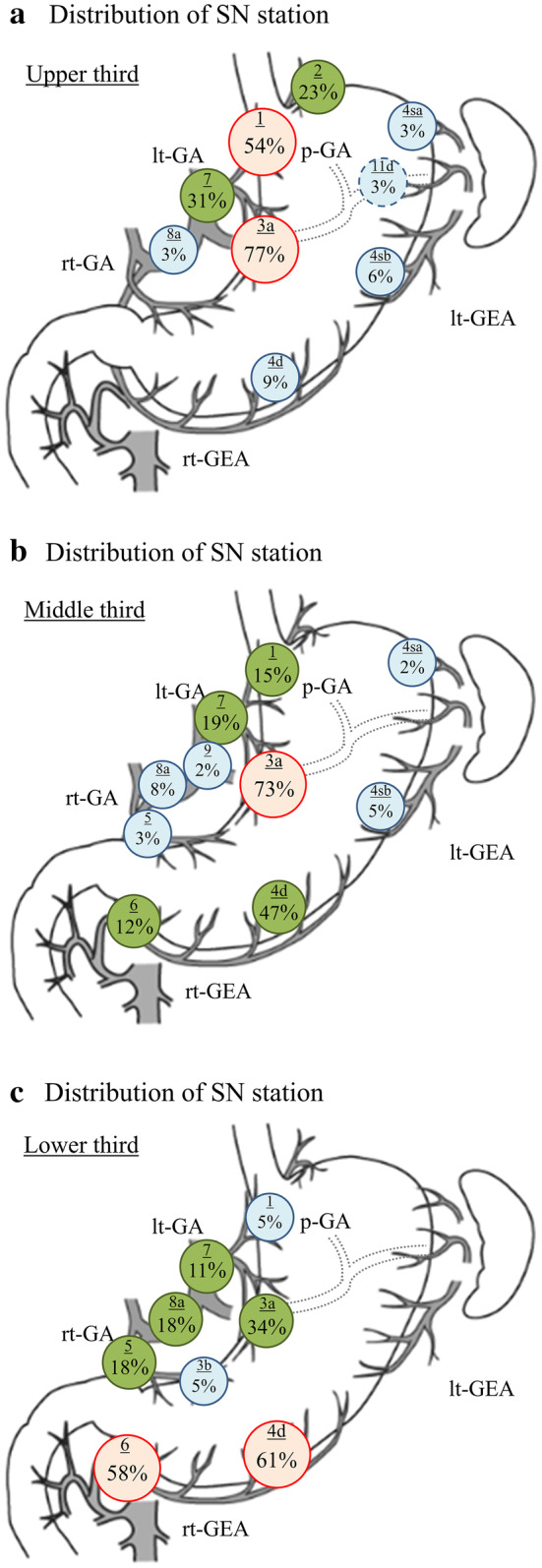
Distribution of the SN stations by a primary tumor location in the long axis of the stomach: **a** upper third. **b** Middle third. SNs were detected in station 3a in > 70% of the cases with a primary tumor at the upper or middle third. Station 4d was also detected as the SN in around half of the cases with a tumor in the middle third. **c** Lower third. Stations 4d and 6 were detected as the SNs in 61% and 58% of the cases, respectively. The red node indicates the stations detected as SNs in > 50% of the cases. The green node indicates the stations detected as SNs between 10 and 50% of the cases. The blue node indicates the stations detected as SNs in < 5%

**Fig. 3:**
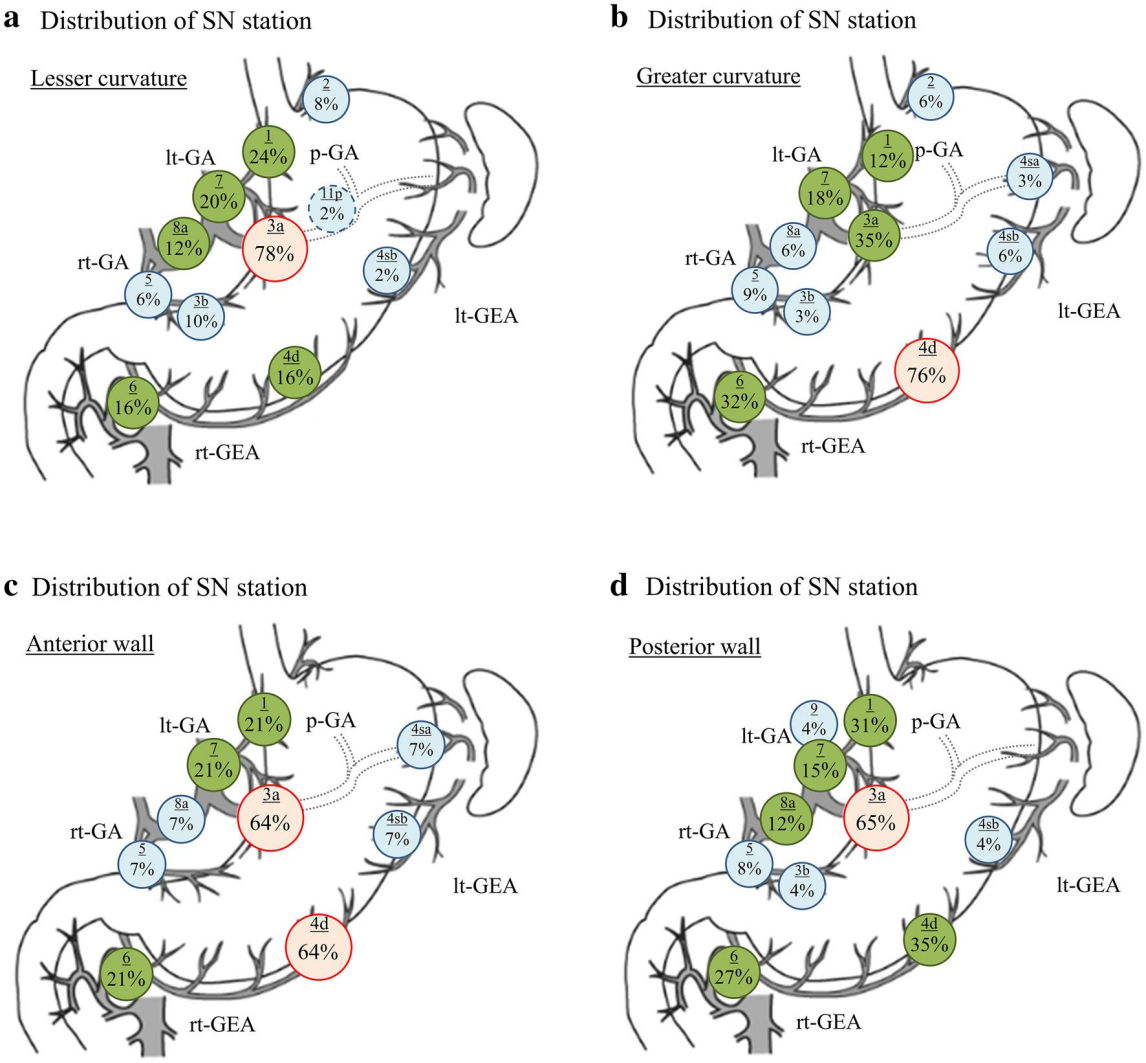
Distribution of the SN stations by a primary tumor location in the short axis of the stomach: **a** Lesser curvature. SNs were detected in station 3a in 78% of cases with a primary tumor located in the lesser curvature. **b** Greater curvature. SNs were detected in station 4d in 76% of cases with a primary tumor located in the greater curvature. **c** Anterior wall. The SNs in stations 3a and 4d were detected in 64% of the cases, respectively. **d** Posterior wall. Station 3a was detected as the SN in 65% of the cases with a tumor located at posterior wall. The red node indicates the stations detected as SNs in > 50% of the cases. The green node indicates the stations detected as SNs between 10 and 50% of the cases. The blue node indicates the stations detected as SNs in < 5%

Finally, the SN distribution by primary tumor location is shown in Figs. 2 and 3. SNs were detected in station 3a in > 70% of cases with a primary tumor in the upper or middle third. Station 4d was also detected as the SN in approximately 50% of the cases with a tumor located at the middle third. On the other hand, in the cases with a tumor located in the lower third, the SNs in stations 4d and 6 were detected in 61% and 58% of the cases, respectively. (Fig. 2) By the tumor location in the short axis of the stomach, SNs were detected in station 3a in 78% of cases with a primary tumor located in the lesser curvature, or in station 4d in 76% of cases with a primary tumor located in the greater curvature. In the cases with a tumor located in the anterior wall, the SNs in stations 3a and 4d were detected in 64% of the cases, respectively. Station 3a was also detected as the SN in 65% of the cases with a tumor located in the posterior wall (Fig. 3). Regarding the SN basin, no statistically significant intergroup difference was found regardless of primary tumor location based on both the long axis and the short axis of the stomach (Table 4).

**Table 4:** Distribution of SN basin

	ER (+) *N* = 132		ER (−) *N* = 275		*p* value
	(%)	95% CI	(%)	95% CI	
Tumor location based on the long axis of the stomach					
Upper					
l-GA	100.0	(100.0 to 100.0)	94.2	(87.7 to 100.8)	0.216
l-GEA	8.8	(− 1.2 to 18.9)	13.5	(3.9 to 23.1)	0.770
r-GA	2.9	(− 3.0 to 8.9)	3.8	(− 1.6 to 9.3)	1.000
r-GEA	11.8	(0.4 to 23.2)	11.5	(2.6 to 20.5)	1.000
p-GA	0	(0.0 to 0.0)	3.8	(− 1.6 to 9.3)	0.363
Others	23.5	(9.0 to 38.6)	13.5	(3.9 to 23.1)	0.361
Middle					
l-GA	86.4	(77.4 to 95.4)	82.3	(75.7 to 89.0)	0.628
l-GEA	6.8	(0.2 to 13.4)	8.5	(3.6 to 13.3)	0.941
r-GA	18.6	(8.4 to 28.9)	9.2	(4.2 to 14.3)	0.117
r-GEA	55.9	(42.9 to 69.0)	54.6	(45.9 to 63.3)	0.993
p-GA	1.7	(− 1.7 to 5.1)	0.8	(− 0.8 to 2.3)	1.000
Others	1.7	(− 1.7 to 5.1)	3.8	(0.5 to 7.2)	0.782
Lower					
l-GA	51.4	(34.6 to 68.2)	66.7	(56.9 to 76.4)	0.156
l-GEA	0.0	(0.0 to 0.0)	1.1	(− 1.13.2)	0.715
r-GA	45.9	(29.1 to 62.8)	34.4	(24.6 to 44.2)	0.306
r-GEA	89.2	(78.7 to 99.7)	79.6	(71.2 to 87.9)	0.296
p-GA	0.0	(0.0 to 0.0)	0.0	(0.0 to 0.0)	NA
Others	0.0	(0.0 to 0.0)	4.3	(0.1 to 8.5)	0.257
Tumor location based on the short axis of the stomach					
Lesser curvature					
l-GA	94.1	(87.7 to 100.6)	92.0	(86.9 to 97.0)	0.625
l-GEA	2.0	(− 1.8 to 5.8)	1.8	(− 0.7 to 4.2)	0.939
r-GA	27.5	(15.2 to 39.7)	22.3	(14.6 to 30.0)	0.477
r-GEA	29.4	(16.9 to 41.9)	34.8	(26.0 to 43.6)	0.496
p-GA	2.0	(− 1.8 to 5.8)	2.7	(− 0.3 to 5.7)	0.784
Others	7.8	(0.5 to 15.2)	9.8	(4.3 to 15.3)	0.685
Greater curvature					
l-GA	52.9	(36.2 to 69.7)	44.2	(30.7 to 57.7)	0.429
l-GEA	8.8	(− 0.7 to 18.4)	17.3	(7.0 to 27.6)	0.267
r-GA	20.6	(6.9 to 34.2)	15.4	(5.6 to 25.2)	0.534
r-GEA	79.4	(65.8 to 93.0)	86.5	(45.9 to 63.3)	0.871
p-GA	0.0	(0.0 to 0.0)	0.0	(0.0 to 0.0)	NA
Others	5.8	(− 2.0 to 13.8)	3.8	(− 1.4 to 9.1)	0.661
Anterior wall					
l-GA	78.6	(57.1 to 100.0)	82.6	(74.0 to 92.2)	0.732
l-GEA	14.3	(− 4.0 to 32.6)	8.7	(0.3 to 12.0)	0.542
r-GA	14.3	(− 4.0 to 32.6)	15.2	(24.6 to 44.2)	0.932
r-GEA	78.6	(57.1 to 100.0)	73.9	(71.2 to 87.9)	0.724
p-GA	0.0	(0.0 to 0.0)	0.0	(0.0 to 0.0)	NA
Others	0.0	(0.0 to 0.0)	0.0	(0.1 to 8.5)	NA
Posterior wall					
l-GA	84.6	(70.7 to 98.5)	83.1	(56.9 to 76.4)	0.858
l-GEA	3.8	(− 3.5 to 11.2)	6.2	(− 1.1 to 3.2)	0.663
r-GA	11.5	(− 0.7 to 23.8)	9.2	(24.6 to 44.2)	0.739
r-GEA	50.0	(30.8 to 69.2)	50.8	(71.2 to 87.9)	0.947
p-GA	0.0	(0.0 to 0.0)	0.0	(0.0 to 0.0)	NA
Others	3.8	(− 3.5 to 11.2)	4.6	(0.1 to 8.5)	0.872

*ER* endoscopic resection, *l-GA* left gastric artery, *l-GEA* left gastroepiploic artery, *r-GA* right gastric artery, *r-GEA* right gastroepiploic artery, *p-GA* posterior gastric artery, *NA* not applicable

## Discussion

The present study demonstrated that SN mapping for patients with gastric cancer after ER showed high detection and diagnostic accuracy rates like in those without previous treatment, although one false-negative case had a non-SN metastasis in the SN basin. Moreover, no significant difference in SN basin distribution was found between the patients after ER and those without previous treatment regardless of the primary tumor location. To the best of our knowledge, this is the first multicenter study with a large cohort to report the feasibility of SN mapping.

The SN detection rate of 98% in the ER group was comparable with that in the non-ER group. When a tracer is injected after ER, the influences of ER, including scarring and ulceration, are concerns. The tracer injection and SN detection should be performed by a physician familiar with SN mapping for gastric cancer. In our study, we had two cases of undetected SN among the patients who underwent the single-tracer method, one with RI and the other with ICG. The SN may be difficult to identify using a single tracer with dye alone in obese patients. As we reported previously, the dual-tracer method is considered the most reliable for identifying the SN in gastric cancer [19, 20]. When considering the tracer type used, a meta-analysis revealed that the SN detection rates in the dye, RI, and dual-tracer groups were 92.1%, 92.1%, and 94.0%, respectively [21]. In addition, the sensitivity, negative predictive value, and accuracy in the dual subgroup were higher than those in the dye and RI groups. Meanwhile, in this study, the SNs could not be identified in a patient in whom perforation occurred during ESD. It may be better not to perform SN biopsy for the perforated cases at least.

Although the dual-tracer method is currently the golden standard, a restriction on use of RI that required a nuclear medicine unit is a matter of concern especially in developing countries. The usefulness of non-radioactive tracer method such as near-infrared observation or magnetic tracer has been reported in recent years. The near-infrared observation using ICG is one of the most promising SN detection methods without RI. By developing the near-infrared imaging system, ICG near-infrared observation can aid in clearly visualizing SN and lymphatic route under laparoscope, making it easy to apply and compatible with laparoscopic surgery. We have also reported the optimal method of ICG near-infrared observation (dose concentration, injection volume, and timing), and its outcomes [22, 23]. In contrast, a practical application of a magnetic tracer completely different from RI is being utilized in case of other cancers. Magnetic tracer using superparamagnetic iron oxide has been used in clinical practice for breast cancer patients [24, 25], although there are no reports of it being used for gastric cancer. If a probe is developed for magnetic tracer that can be used in laparoscopic surgery, it may be used as a promising alternative to RI. These tracers have the potential to become an alternative to dual tracers in the future. Further investigations about non-radioactive tracers are needed to spread the practicability of SNNS for gastric cancer to a wide extent.

Although no change in the lymphatic basin was observed from before to after ER, non-SN metastasis within the SN basin was observed in one of the nine patients with lymph node metastasis in this study. This 11% false-negative rate should not be ignored. Few reports have described detailed examinations of lymph flow after ER. We studied lymphatic flow after ER in a pig model [26]. The evaluation of lymph flow on ICG imaging before and 4 weeks after ESD showed no change in 10 (83.3%) of the 12 cases. However, the pre-ESD lymph flow partially disappeared, and new lymph flow appeared in the lesser curvature side of the middle and lower stomach. Although lymph flow does not generally change with ER, the effect of ER may vary among sites in the long- and short-axis directions. The SN basin reportedly has ≥ 2 complex structures in the middle and lower parts of the stomach as compared with the upper part of the stomach [27]. In the false-negative case, the lymph flow might have been changed by ER. Furthermore, it might have been caused by an SN detection procedure because SN mapping was performed using the only dye method at laparotomy in that case. In the additional surgery for post-ER gastric cancer, whose lymph node metastasis rate is not so high, SNs should be reliably identified to minimize the occurrence of false negatives. In addition, the curability can be ensured by resecting the SNs and SN basin. At least now, validation by a prospective study using the dual-tracer method for post-ER gastric cancer is necessary.

This study has some limitations. The SN detection method was not uniform, as single or dual tracers, and RI or/and dye were used. The investigators from the participating hospitals in this retrospective study conducted a multicenter prospective trial of SN navigation surgery for gastric cancer; therefore, the detailed procedures for SN detection have been unified.

In summary, our findings clarify the feasibility of SN mapping based on the SN basin concept in patients with gastric cancer who previously underwent ER. The multicenter prospective trial of laparoscopic sentinel basin dissection after ESD for early gastric cancer (SENORITA 2 trial [28]) is ongoing in Korea. We are also planning to start a prospective trial using the dual-tracer method to validate the SN concept for post-ER gastric cancer.
